# Impact of Lower Strength Alcohol Labeling on Consumption: A Randomized Controlled Trial

**DOI:** 10.1037/hea0000622

**Published:** 2018-04-26

**Authors:** Milica Vasiljevic, Dominique-Laurent Couturier, Daniel Frings, Antony C. Moss, Ian P. Albery, Theresa M. Marteau

**Affiliations:** 1 Behaviour and Health Research Unit, Institute of Public Health, University of Cambridge; 2 Centre for Addictive Behaviours Research, Division of Psychology, School of Applied Sciences, London South Bank University; 3 Behaviour and Health Research Unit, Institute of Public Health, University of Cambridge

**Keywords:** lower strength alcohol labeling, consumption, bar lab, randomized controlled trial

## Abstract

***Objective:*** Labels indicating low/light versions of tobacco and foods are perceived as less harmful, which may encourage people to consume more. There is an absence of evidence concerning the impact on consumption of labeling alcohol products as lower in strength. The current study tests the hypothesis that labeling wine and beer as lower in alcohol increases their consumption. ***Method:*** Weekly wine and beer drinkers (*n* = 264) sampled from a representative panel of the general population of England were randomized to one of three groups to taste test drinks in a bar-laboratory varying only in the label displayed; *Group 1*: verbal descriptor *Super Low* combined with 4% alcohol by volume (ABV) for wine/1% ABV for beer; *Group 2*: verbal descriptor *Low* combined with 8% ABV for wine/3% ABV for beer; *Group 3*: no verbal descriptors of strength (*Regular*). Primary outcome was total volume (ml) of drink consumed. ***Results:*** The results supported the study hypothesis: the total amount of drink consumed increased as the label on the drink denoted successively lower alcohol strength, B_Lin_ = .71, *p* = .015, 95% CI [0.13, 1.30]. Group contrasts showed significant differences between those offered drinks labeled as *Super Low* (*M* = 213.77) compared with *Regular* (*M* = 176.85), B = 1.43, *p* = .019, 95% CI [0.24, 2.61]. There was no significant difference in amount consumed between those offered drinks labeled as *Low* compared with *Regular*. ***Conclusions:*** These results suggest that labeling drinks as lower in strength increases the amount consumed. Further studies are warranted to test for replication in non-laboratory settings and to estimate whether any effects are at a level with the potential to harm health. ***Trial Registration:*** ISRCTN15530806.

Alcohol is the fifth leading cause of mortality and morbidity both in the United Kingdom and globally (Burton et al., 2017; Sassi, 2015). One approach to reducing alcohol consumption and associated harms is the development, promotion, and marketing of lower alcohol products (Department of Health, 2012). Current legislation across the European Union (EU) limits the use of low/er alcohol descriptors to drinks of 1.2% alcohol by volume (ABV) and lower, with similar restrictions found globally (see Canadian Food Inspection Agency, 2017; Food Standards Australia & New Zealand, 2014; The European Parliament and the Council of the European Union, 2011). Current regulations surrounding the restricted use of terms denoting lower alcohol to promote such products will end in 2018 providing a timely opportunity to consider whether extending the range of alcohol strengths to which they can be applied can contribute to policies designed to reduce alcohol consumption across the population. This interest is captured in the most recent U.K. Government Alcohol Strategy published in March, 2012, that, among other policies, included an industry pledge through the Responsibility Deal to take one billion units of alcohol out of the market by 2015, primarily through increasing consumer selection of lower alcohol products (Department of Health, 2012).

One of the strategies considered to reduce the alcohol units on the market is to allow the industry to use a wider variety of low/er strength alcohol labels to promote more widely products with alcohol content lower than the current average on the market (U.K. averages: 12.9% for wine and 4.2% for beer; Department of Health, 2014), but higher than the currently legislated limit of 1.2% ABV. While current sales data show that the alcohol market is dominated by regular/standard (average) strength products (Department of Health, 2014), recent years have seen a growing interest from consumers in lower strength and no-alcohol products. This is especially noticeable in high income countries such as the United Kingdom, USA, Canada, and Germany (“Big brewers see strong potential for weak beer,” 2016; Wine Intelligence, 2013). Increasing consumer selection of lower strength alcohol products in place of regular strength products forms part of a wider policy approach to regulate the availability of alcohol (including physically restricting density of outlets, reducing the hours and days of sale, regulating the minimum legal purchase age, and offering different availability by alcohol strength; for comprehensive reviews of this and other alcohol policies see Babor et al., 2010; Burton et al., 2017).

For lower strength alcohol products to reduce consumption depends upon these products being selected in place of equal volumes of higher strength products as opposed to simply increasing the number of occasions perceived suitable for alcohol consumption (see also Anderson & Rehm, 2016; Rehm, Lachenmeier, Llopis, Imtiaz, & Anderson, 2016; Vasiljevic, Coulter, Petticrew, & Marteau, 2018). To achieve this, such labels must not engender a self-licensing effect, that is give people permission to consume more when given what might be interpreted as a virtuous product. In the current context, a lower strength alcohol product might be seen as virtuous and, if it leads to self-licensing could result in consumption of more alcohol than would have been consumed from a higher strength product alone (Khan & Dhar, 2006; Shemilt, Hendry, & Marteau, 2017).

A recent systematic review by Shemilt and colleagues (2017) summarizing studies of product labeling denoting low content in food (*k* = 19) and tobacco (*k* = 6), found the potential for self-licensing effects by showing that low content labels can alter people’s perceptions concerning the content of products, and what they judge to be an appropriate serving (in food). However, there was an absence of evidence regarding the behavioral impact of such labels, and no studies on alcohol content labeling were identified.

The aim of the present study is to assess the impact of lower strength alcohol labeling on consumption and test the hypothesis that labeling alcohol products to denote lower levels of alcohol by volume increases consumption. For the purposes of this study we used two different labels denoting lower alcohol strength. *Low* and *Super Low* are the two verbal descriptors denoting lower alcohol strength used in the current study, based on a previous study in which these were found to be the terms that most differentiated alcohol products of low and lower strengths (Vasiljevic, Couturier, & Marteau, 2018). In this initial study, we found that a sample of 1,600 weekly wine and beer drinkers from the U.K. population, perceived the verbal descriptors *Low, Lower, Light, Lighter,* and *Reduced* as similar and lower in strength (wine: 6.7–8.3%, beer: 2.7–3.1%) than the descriptor *Regular* (average % ABV), but higher in strength than the descriptors *Extra Low, Super Low, Extra Light,* and *Super Light* (wine: 3.5–4.8%, beer: 1.3–2.2%). These latter descriptors were perceived as similar (see Vasiljevic, Couturier et al., 2018). Furthermore, among the two perceptual clusters, the descriptors *Low* and *Super Low* were the most differentiated and were thus used in the current study.

In the present research, the verbal descriptor *Low* was coupled with 8% ABV in wine and 3% ABV in beer. The verbal descriptor *Super Low* was coupled with 4% ABV in wine and 1% ABV in beer. In addition to these values corresponding to the perceptions of strength in the previous study, we judged them to also have the greatest face validity. The two lower strength labels were contrasted to a *Regular* label, denoting the average % ABV available in the United Kingdom (12.9% for wine and 4.2% for beer), with no verbal descriptor of strength (which is how they are presented for sale). Only the labels placed against the drinks differed between participants, according to randomization, with all other variables held constant.

## Method

### Design

A between-subjects experiment with one independent factor of three levels corresponding to the label that accompanies drinks (wine or beer) for consumption. Participants were assigned to taste either wine or beer according to stated prior preference at recruitment. They were then randomly allocated to one of three groups to taste test three glasses of their preferred drink, with all three glasses having one of three possible labels—*Group 1*: label displaying the verbal descriptor *Super Low* combined with either 4% ABV for wine or 1% ABV for beer; *Group 2*: label displaying the verbal descriptor *Low* combined with either 8% ABV for wine or 3% ABV for beer; or *Group 3*: label displaying no verbal descriptor of strength and showing the average % ABV of the drink currently on sale in the United Kingdom, corresponding to a standard/regular drink: 12.9% for wine and 4.2% for beer.

### Participants

Two hundred and sixty-four weekly wine and beer drinkers were sampled from an existing representative panel of the general population of England. The representative panel was provided by a market research agency (https://www.icmunlimited.com/). According to prior stated preference, we sampled 132 wine drinkers and 132 beer drinkers. Participant inclusion criteria included healthy adults (above 18 years of age), weekly consumption of alcohol, and a preference for either wine or beer. Exclusion criteria included pregnancy (women only), current medication use (including antibiotics), and a history of neurological or psychiatric disorders. Eligible weekly wine and beer drinkers taste tested either wine or beer according to their drink preference. Once their eligibility for the study and drink preference was ascertained, they were randomized to one of the three experimental groups varying in the labels used to describe the drinks they were invited to taste, but not in the actual drinks (see Design).

Within the two groups of participants that preferred wine and those that preferred beer, we stratified randomization to the three experimental groups by setting interlocking quotas for age, gender, and socioeconomic status (SES). SES classification was based on an index of occupational status using the U.K. Registrar General’s social classification with participants divided into three SES groups (see Rose & Pevalin, 2001): higher managerial and professional [high], white collar and skilled manual [medium], and semi-skilled and unskilled manual [low]. Setting these quotas allowed us to obtain roughly equal numbers of participants of different ages, genders, and SES status across the three experimental groups in order to explore the moderating effects of these variables on the effect of lower strength alcohol labeling. Within each interlocking quota block (comprising a combination of type of drink, age, gender and SES), participants were randomized to one of the three experimental groups by means of an algorithm implemented using R software by the study statistician (DLC) before recruitment of participants commenced.

The randomization allocation to experimental group was concealed from the market research agency recruiters who assigned participants to a unique participation number according to their drink preference, age, gender, and SES membership. Participants were blinded to assignment of experimental group (open-ended questions at the end of the testing session confirmed that participants were not aware of the study hypotheses and were not aware that the labeling of alcohol strength was manipulated across different groups). For demographic and other individual difference characteristics of our sample see Table 1 below. Randomization was successful; there were no significant differences between the three experimental groups on any of the characteristics. The final sample size of 264 participants provided 90% power at 5% level of significance to detect a medium sized effect (0.5 *SD*) in consumption between the *Regular* label (no verbal descriptor of strength given) and one of the “low alcohol” (*Super Low* or *Low*) labels. Our power calculations were based on studies using a similar taste-preference task design carried out in the same bar lab setting with medium effect sizes, which examined consumption of placebo drinks in participants exposed to a Drinkaware poster versus a control condition (Moss et al., 2015).[Table-anchor tbl1]

### Measures

#### Primary outcome

Total volume of drink consumed (in ml) was the primary outcome. The total volume of drink poured into each glass was measured using high precision scales (Smart Weigh Model PL11B). In order to ascertain how much of the drinks participants consumed, the liquid remaining in the glasses at the end of the study period was measured using the same scales and subtracted from the initial total volume poured into the glasses.

#### Secondary outcomes: Product appeal

Two items measured participants’ appeal of the product they saw: “How likely are you to buy this wine/beer?” and “How likely are you to drink this wine/beer?” (both items were answered on scales running from *1 = very unlikely* to *7 = very likely*), *r* = .87, *p* < .001.

#### Secondary outcomes: Understanding of alcohol strength

Three items measured participants’ understanding of the alcohol strength of the product. The first item gauged participants’ knowledge of whether the wine/beer they saw could be safely consumed by children: “This wine/beer can be safely drunk by children aged over 12. Do you agree with this statement?” Responses were recorded on a scale from *1* = *strongly disagree* to *7* = *strongly agree*. Participants’ responses were dichotomized whereby any level of disagreement with the statement was considered correct, and any level of agreement as incorrect.

The second item gauged participants’ understanding of how many drinks of the wine or beer they could have without exceeding the drink-driving legal limit: “How many small glasses (125 ml) of this wine/half pints of this beer do you think you could have and still drive within the legal limit?” Responses were recorded on a 0–20 slider. To determine the accuracy of participants’ responses, we calculated how many half pints of beer or small glasses (125 ml) of wine participants could drink and still drive within the legal driving limit for the United Kingdom (excluding Scotland). This was done for all the different levels of % ABV, compiling scores separately for men and women, and based on a person with average weight and metabolism (for more details on the calculations, see the online supplementary materials).

The third item gauged participants’ understanding of unit content of the drink they were shown: “How many units of alcohol do you think a small glass (125ml)/half pint of this wine/beer would have?” Responses were recorded on a 0–20 slider. For analysis we determined the actual number of units contained in each of the drinks according to its purported % ABV (see online supplementary materials).

#### Secondary outcomes: Calorie content

Perception of the calorie content of the presented drink was assessed by one item: “The recommended daily calorie intake from food and drinks for men is 2,500 calories (kcal), and for women 2,000 calories (kcal). How many calories (kcal) do you think a small glass (125ml)/half pint of this wine/beer has?” Responses were open-ended, but constrained to responses ranging from 0 to 2,500.

#### Secondary outcomes: Guilt associated with consumption

One item based on Wansink and Chandon (2006): “How guilty would you feel after consuming a small glass (125ml)/half pint of this wine/beer?” Answers were recorded on scales from *1 = not guilty* to *9 = guilty*.

#### Other measures: Risky drinking

This was assessed using the AUDIT-C (Bush, Kivlahan, McDonell, Fihn, & Bradley, 1998), which comprises the first three items of the Alcohol Use Disorders Identification Test (AUDIT; Babor, Higgins-Biddle, Saunders, & Monteiro, 2001). A sample item asked “How many drinks containing alcohol do you have on a typical day when you are drinking?” with response options ranging from *1 or 2, 3 or 4, 5 or 6, 7 to 9, 10 or more*. Following recommendations by Public Health England (2017), responses to the AUDIT-C were summed and dichotomized to denote riskier (scoring above 5) versus less risky drinking patterns (scoring below 5).

#### Other measures: Motivation to reduce consumption

Three items were used to measure intentions and desire to drink less within the next six months: “Thinking about the next 6 months: I intend to drink less alcohol/I want to drink less alcohol/I will try to drink less alcohol” Responses were recorded on 7-point scales ranging from *1 = strongly disagree* to *7 = strongly agree,* Cronbach’s alpha = .93.

#### Other measures: Self-licensing

Two items assessed “self-licensing” that is, participants’ self-reported deservingness to act indulgently following what might be interpreted as a virtuous choice: “If I were to have a low alcohol drink, I would feel like I deserved to have something stronger for my next drink” and “If I were to have a low alcohol drink, I would feel like I could have more than my usual number of drinks.” The items were rated on 7-point scales ranging from *1 = strongly disagree* to *7 = strongly agree, r* = .34, *p* < .001.

#### Other measures: Demographic characteristics

The following were recorded: *age, gender, ethnicity*, and *SES* (assessed using individual-level measures of highest educational qualification, income and occupational status, and area-level (i.e., neighborhood) deprivation assessed from postcode information and transformed into an Index of Multiple Deprivation, IMD; see Oguz, Merad, & Snape, 2013). The IMD is the official measure of relative deprivation for small areas (or neighborhoods) in England, which ranks every small area in England from 1 (most deprived area) to 32,844 (least deprived area). This ranking is then transformed into either quintiles or deciles of area-level deprivation for use in analyses.

### Procedure

**Figure 1 fig1:**
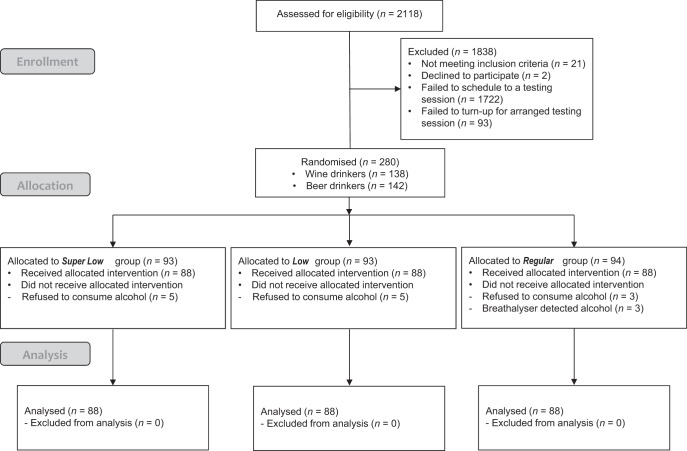
CONSORT diagram of participant flow through the study.

The study was approved by the University of Cambridge’s Psychology Research Ethics Committee [PRE.2015.083], and the London South Bank University Research Ethics Committee [UREC 1468]. The flow of participants through the study can be seen in the CONSORT flow diagram below (see Figure 1).[Fig-anchor fig1]

The study was conducted in a laboratory setting mimicking a bar environment, located within a university psychology department in England. The bar lab is a purpose-built testing room resembling a typical pub environment, featuring a 4.5 m bar, optics, bar taps, bottles, a slot machine, bar stools, and appropriate wall hangings (see Figure S1 in online supplementary materials). Testing took place during weekdays in 30-min slots between 12.00 and 20.00. Recruitment took place from November 2016 to March 2017. Participants were recruited from an existing nationally representative panel. Participants were contacted via e-mail and telephone by a market research agency recruiter who ascertained their eligibility for the study (see also Participants section).

Upon arrival at the bar lab, participants were told they were undertaking a taste-preference task in which they would rate the quality of different alcoholic beverages. Participants then provided their written consent to participate in the study, at which point they were breathalyzed with a Lion Alcometer 600 (Lion Laboratories, Barry, U.K.). Anyone testing positive (above 0 breath alcohol concentration, BrAC) on the breathalyzer was deemed ineligible and stopped from further participation (see CONSORT Flow in Figure 1).

Participants first took part in a sham taste-preference task, which served as a cover story and allowed us to gauge participants’ consumption of the beverages without revealing the true purpose of the study (see Moss et al., 2015; Stautz, Frings, Albery, Moss, & Marteau, 2017). The taste-preference task is a validated method for assessing alcohol consumption in laboratory studies, which has also been validated as an analogue for participants’ real-world alcohol use outside of the lab (see Jones et al., 2016). Participants were seated at the bar-counter during the taste test. To avoid possible ceiling effects, wine drinkers were provided with three glasses of wine with 125 ml in each glass (strength 5.5% ABV), and beer drinkers were provided with three glasses of beer with 250 ml in each glass (strength 2.8% ABV). The labels comprised small pieces of card placed in front of the glasses. The study used a between-subjects design, which necessitated all three glasses to be labeled with the same label. A cover story was therefore used purporting that the three glasses of wine or beer came from the same producer, used the same ingredients, but were fermented in vessels made from different materials, which can affect taste. Thus, for example, a participant who preferred wine and was randomized to experimental Group 2, which denoted a *Low* strength label (see Design) would have been presented with three glasses of wine all labeled as *Low* 8% ABV, with the cards in front of each glass purporting to be from different fermentation vessels A, B, or C. The task of the participants was to rate the three samples A, B, and C (see Figure S2 in online supplementary materials for an example set-up of the taste test in one experimental group). A glass containing 250 ml of water was available as a palate cleanser. Participants were asked to rate how pleasant, strong tasting, sweet, and fizzy the drinks are (adapted from Field & Eastwood, 2005; see online supplementary materials for the full instructions regarding the taste test). Participants were told they could drink as much or as little as they liked to make their ratings and were informed that the taste test lasts 10 min. The experimenter remained in the bar laboratory for the duration of the taste test.

After the taste test, the drinks were removed, and the participants were given a second questionnaire which contained the secondary outcomes, and the demographic and individual difference measures. At the end of the study procedures, participants were debriefed about the true nature of the study, and we revealed that all the drinks tasted were of “lower alcohol” strength including in the condition where the labels purported the drinks to be of regular strength. At this point, participants underwent another breathalyzer test to gauge their intoxication. Participants who were above the English driving limit (35 micrograms of alcohol per 100 milliliters of BrAC) were asked to stay in the lab until the effects of the alcohol had dissipated or to take public transportation when leaving the lab. Once participants left the bar laboratory, the fluid they did not consume was measured (allowing a calculation of fluid consumed). Participants were reimbursed with a 30-pound (circa 37 USD) check for their participation. The trial protocol was registered with the ISRCTN registry and can be accessed via the following reference number ISRCTN15530806.

### Analysis

Multiple linear and logistic regression analyses were used with linear trends (linear trend: −1 = *Regular*, 0 = *Low*, +1 = *Super Low*) to examine whether reduced levels of alcohol strength as denoted by labels was associated with a linear increase in consumption and a linear change in self-reported indices of appeal and understanding of strength. To understand differences between lower and regular labels, we performed contrast analyses by regressing the transformed consumption data on two dummy variables representing the experimental conditions (*Low*: D_1_ = 1, D_2_ = 0; *Super Low*: D_1_ = 0, D_2_ = 1; *Regular*: D_1_ = 0, D_2_ = 0), and a dummy variable denoting the type of drink (wine: D_3_ = 0; beer: D_3_ = 1), using percentile bootstrapping with 5,000 resamples to derive parameter estimates. We applied a Holm-Šídák correction to adjust alpha for the familywise error; all significant comparisons exceeded the adjusted alpha level.

## Results

### Primary Outcome

An examination of the data revealed five univariate outliers who consumed more than 605 ml (> ¯*x* + 3SD) of fluid in total; therefore, these outliers were substituted with the next highest value in the distribution 588 ml (Tabachnick & Fidell, 2013). The data for the primary outcome were positively skewed; hence, we performed a square root transformation of the data, which approximated the distribution to normal. Untransformed data are provided for descriptive statistics for clarity.

**Figure 2 fig2:**
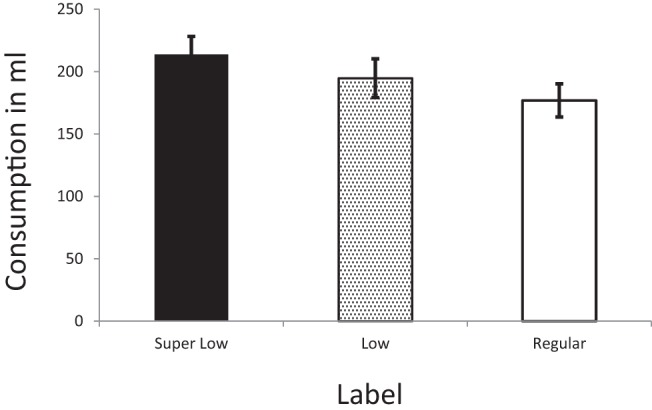
Graphical presentation of consumption levels across the three experimental groups.

The results showed a significant linear trend whereby the total amount of drink consumed increased as the label on the drink denoted successively lower alcohol strength, B_Lin_ = .71, *SE* = .30, *p* = .015, 95% CI [0.13, 1.30] (see Figure 2). Planned contrasts revealed that participants drank more beer (*M* = 249.19, *SD* = 139.41) than wine (*M* = 140.96, *SD* = 84.31), B_D3_ = 3.77, *SE* = .51, *p* < .001, 95% CI [2.78, 4.76]. Participants also drank more when the drinks were labeled as *Super Low* alcohol strength (*M* = 213.77, *SD* = 124.05) when compared with the drinks labeled as *Regular* (*M* = 176.85, *SD* = 116.41), B_D2_ = 1.43, *SE* = .61, *p* = .019, 95% CI [0.24, 2.61]. In contrast, participants’ consumption of drinks labeled as *Low* alcohol strength (*M* = 194.60, *SD* = 138.65) did not differ from participants’ consumption of drinks labeled as *Regular* (*M* = 176.85, *SD* = 116.41), B_D1_ = .59, *SE* = .63, *p* = .340, 95% CI [−0.66, 1.80]. See Table 2 and Table 3 for the full models described above. We performed several sensitivity tests (a) by controlling for total consumption of water during the taste test; (b) by using *z* standardized scores to indicate the amount consumed for wine and beer; and (c) running a robust regression on the raw untransformed data (Heritier, Cantoni, Copt, & Victoria-Feser, 2009), which yielded the same pattern of results as reported above. [Fig-anchor fig2][Table-anchor tbl2][Table-anchor tbl3]

We repeated the above analysis while adding gender (female: D_4_ = 0; male: D_4_ = 1), age (18–44 years: D_5_ = 0, 45–70 years: D_5_ = 1), SES occupational status (linear trend: −1 = low, +1 = high) as predictors (i.e., main effects) and as moderators (i.e., 2-way interactions) of (a) the linear trend and, in separate analyses, (b) the contrasts of the experimental conditions (*Low*: D_1_ = 1, D_2_ = 0; *Super Low*: D_1_ = 0, D_2_ = 1; *Regular*: D_1_ = 0, D_2_ = 0). As in the primary models, the effect of drink persisted: participants drank more beer than wine overall. In addition, there was a significant main effect of gender, whereby men (*M* = 237.28, *SD* = 136.87) drank more compared with women (*M* = 152.86, *SD* = 100.60), when (a) the linear trend of experimental groups was entered in the model: B_D4_ = 1.90, *SE* = .60, *p* = .002, 95% CI [0.74, 3.07]; and (b) when contrasts between experimental groups were examined: B_D4_ = 2.73, *SE* = .94, *p* = .004, 95% CI [0.86, 4.58]. The linear trend of label, B_Lin_ = 1.05, *SE* = .43, *p* = .016, 95% CI [0.18, 1.93] remained unaltered, as did the contrast between the *Super Low* and *Regular* label, B_D2_ = 2.11, *SE* = .87, *p* = .016, 95% CI [0.34, 3.79]. While the effect sizes were unaltered, they were rendered statistically nonsignificant when applying a Holm-Šídák multiplicity correction due to the large number of predictors included in the model. No other effects were statistically significant after multiplicity correction.

We repeated the above analysis including SES education (linear trend: −1.5 = lowest quartile, +1.5 = highest quartile); SES income (linear trend: −1.5 = lowest quartile, +1.5 = highest quartile); SES index of multiple deprivation (linear trend: −2 = lowest quintile, +2 = highest quintile); risky drinking (not risky: D_8_ = 0; risky: D_8_ = 1); motivation to reduce consumption (continuous, centered); and self-licensing (continuous, centered) as predictors and as moderators of (a) the linear trend and (b) the contrasts of the experimental conditions. The effect sizes of drink and gender observed in the previous models remained unaltered, but were no longer statistically significant after applying multiplicity corrections due to the larger number of predictors. Similarly, the linear trend of label, B_Lin_ = 2.23, *SE* = 0.80, *p* = .004, 95% CI [0.72, 3.80] was unaffected, as was the contrast between the *Super Low* and *Regular* label, B_D2_ = 4.67, *SE* = 1.66, *p* = .004, 95% CI [1.62, 8.10]. As before, these effects were rendered statistically nonsignificant after applying the Holm-Šídák multiplicity correction. The only significant effect that remained *after* multiplicity correction was a main effect of risky drinking: risky drinkers drank more than nonrisky drinkers, B_D8_ = 2.46, *SE* = 0.72, *p* = .001, 95% CI [1.00, 3.83]. However, this effect only emerged in the regression modeling differences between experimental conditions as a linear trend. Appendix 4 in the online supplementary materials contains tables showing the full models fitted described above.

### Secondary Outcomes

Product appeal was negatively skewed so we performed a logarithmic transformation on the inversed scores, which approximated the distribution to normal. We repeated the same regression analysis described above, which yielded no significant results. Adding age, gender, and SES occupational status to the model as predictors and as moderators of the linear trend and the contrasts also yielded no significant effects.

#### Understanding of alcohol strength and calorie estimation

The items gauging participants’ understanding of alcohol strength and calorie estimation were transformed so that participants’ ratings were compared with the factually correct answer (see online supplementary materials for all transformations and graphical presentation of the results). When modeling the log odds of the correct understanding of strength, logistic regressions showed no significant linear trend, nor significant contrasts in understanding whether a drink is appropriate for consumption by children, with all participants displaying a similarly high understanding regardless of the drink label (%_SuperLow_ = 71; %_Low_ = 77; %_Regular_ = 75).

There was a significant linear trend in the level of understanding of the drink-drive limit; participants’ understanding increased as the rate of alcohol strength decreased, B_Lin_ = 17.81, *SE* = .40, *p* < .001, 95% CI [16.85, 18.43]. Planned contrasts revealed that participants who saw the *Super Low* and *Low* labels (%_SuperLow_ = 100; %_Low_ = 100) were more accurate when compared with participants seeing the *Regular* label (%_Regular_ = 89), B_D2_ = 19.15, *SE* = .39, *p* < .001, 95% CI [18.21, 19.74]; B_D1_ = 19.15, *SE* = .39, *p* < .001, 95% CI [18.23, 19.75].

There was also a significant linear trend in the level of understanding of unit content in the different drinks, whereby participants’ underestimation of the alcohol units increased with increased alcohol strength, B_Lin_ = 1.15, *SE* = .24, *p* < .001, 95% CI [0.74, 1.69]. Planned contrasts revealed that participants seeing the *Super Low* label (%_SuperLow_ = 99) were significantly more accurate at unit estimation when compared with those randomized to the *Regular* label condition (%_Regular_ = 75), B_D2_ = 3.37, *SE* = 8.24, *p* = .002, 95% CI [2.03, 20.48]. Those seeing the *Low* label fell in between these two conditions (%_Low_ = 84), though the contrast between the *Low* and *Regular* label did not reach significance, *p* = .140.

There was no statistically significant linear trend in the estimation of calories; however, contrast analyses revealed that participants seeing the *Low* label (%_Low_ = 94) were less likely to underestimate the amount of calories when compared with those in the *Regular* label condition (%_Regular_ = 80), B_D1_ = 1.45, *SE* = 1.39, *p* = .003, 95% CI [0.53, 2.98]. Those seeing the *Super Low* label fell in the middle (%_Low_ = 90), though the contrast between the *Super Low* and *Regular* label did not reach significance, *p* = .064.

#### Guilt

There were no significant linear trends nor significant contrasts between the label conditions in the amount of self-reported guilt attached to consuming the given drinks.

## Discussion

Participants drank most when drinks were labeled as *Super Low* and least when labeled as *Regular* strength. Age, gender, SES, risky drinking, motivation to reduce consumption, and self-licensing did not moderate these effects. There were no significant differences in appeal between the different experimental conditions. Understanding of alcohol strength and calorie content was generally good.

We tested participants in a bar laboratory enabling us to have strict control over the testing environment. However, the bar laboratory is still a laboratory setting housed in a university department, and therefore, the consumption behaviors displayed by participants may not fully reflect how drinkers would respond to lower strength alcohol labeling in real-world drinking settings, such as actual bars, restaurants, public events (gigs, concerts, sporting events), as well as off-license settings such as in the home. Future replications should therefore test the effects of lower strength alcohol labeling in these real-world environments.

Our primary outcome was measured as part of a 10-min taste test, a commonly used validated measure in laboratory studies of alcohol consumption (Jones et al., 2016). Nonetheless, naturalistic consumption of alcohol differs from this in several ways that might affect consumption of different strengths of alcohol. Time is one such difference. The time taken to consume a drink is usually longer than 10 min. It is possible that the observed greater consumption of drinks labeled as lower strength may only be apparent over a short time-period if drinkers pace their intake of the higher strength alcohol, with the lower strength alcohol being consumed at a faster pace (see Higgs, Stafford, Attwood, Walker, & Terry, 2008). However, since participants across all three experimental groups were given the same alcoholic drink with alcohol strength held constant while only varying the labels presented, we could reject the hypothesis that participants paced their drinking rate and consumption because of the pharmacological cues they could detect in the drinks as in the Higgs and colleagues’ study (2008). Nevertheless, future studies should extend the current research to incorporate longer testing periods, while also examining other relevant outcomes such as consumption duration, sip-rate, and sip-duration in order to better understand the impact of lower strength alcohol labeling.

Furthermore, while people may drink more if drinks are labeled as lower in strength, we do not know if this is sufficient to result in the consumption of more units of alcohol overall from lower strength alcohol drinks. Our study was not set up to test this since we held the strength of the alcohol constant across experimental groups. Future studies should manipulate the alcohol strength in conjunction with manipulating the labels to examine this question.

This is the first study to examine the impact of lower strength alcohol labeling on actual consumption. The findings from this study have important ramifications for current discussions between industry and policymakers who are interested in reducing the total level of alcohol consumed by extending the % ABV range of products that are allowed to carry lower strength alcohol labels beyond the currently legislated cap of 1.2% ABV (Department of Health, 2012). We found that participants consumed successively greater amounts of wine and beer when labels communicated successively lower alcohol strengths consistent with a self-licensing effect. These findings suggest that further research is needed to determine whether policies extending the range of lower strength alcohol labels could have unintended consequences such as increasing the total amount of alcohol consumed in the policy jurisdiction (Khan & Dhar, 2006; Shemilt et al., 2017). Despite a clear interest from the industry and policymakers to extend the range of % ABV and the verbal descriptors that could be used to denote lower alcohol strength on alcohol labels (Department of Health, 2012), little is known about the ironic effects on consumption revealed in the present research (see also Anderson & Rehm, 2016; Rehm et al., 2016; Vasiljevic, Coulter, et al., 2018). Since this is the first study to examine this issue, replications and extensions are needed to ascertain the potential for lower strength alcohol labeling to exert a paradoxical effect that is detrimental to public health.

Importantly, although there was a significant linear trend whereby participants drank successively more alcohol volume with decreasing label strength, the contrast between the *Low* and *Regular* groups did not reach statistical significance. This may reflect a true null effect or insufficient power to detect a smaller effect size. From a policy perspective, it will be important for future studies to have sufficient power to estimate smaller effect sizes given more products are likely to be labeled *Low* as opposed to *Super Low*, and given that the potential harm arising from overconsumption of such products would be greater from products of *Low* strength as opposed to products of *Super Low* strength.

We also examined whether demographic characteristics or the assessed individual difference variables affected alcohol consumption. Gender and risky drinking (as measured by the AUDIT-C) predicted alcohol consumption: men and those classified as riskier drinkers consumed more alcohol. However, neither gender nor risky drinking moderated the effects of lower strength alcohol labeling on consumption, suggesting that different types of drinkers were not differentially affected by the labels. None of the other demographic or individual difference variables measured in the present study moderated the effects of labeling on alcohol consumption. These initial findings therefore suggest that lower strength alcohol labeling may have similar effects across different groups in the population. To provide more robust evidence for a null effect, future studies should also test for moderating variables, in studies with larger sample sizes and examining more diverse groups of participants.

In the present research, there were no significant differences in levels of self-reported appeal. This is not surprising since participants in all three experimental groups were given the same wine/beer with only the labels differing between participants. If anything these effects speak to the successful manipulation of labeling while keeping the drinks constant, which allowed us to control for possible confounding influences that may have arisen if we used different drinks across the different groups. Nevertheless, future research could examine differential effects of manipulating both the drinks’ labels and the drinks themselves.

The dissociation between self-reported appeal and actual consumption of the drinks could indicate that labeling of lower alcohol strength impacts people’s behaviors largely via implicit processes without conscious awareness (see Strack & Deutsch, 2004). This suggests that labels that do not highlight the lower alcohol content of drinks may be more effective in reducing consumption than those in which the lower alcohol content is highlighted (in line with prior findings by Geller, Kalsher, & Clarke, 1991). This hypothesis merits testing.

Understanding of alcohol strength and calorie content was generally high across experimental conditions with the majority of participants correctly identifying or erring on the side of caution regarding consumption of the products by children aged over 12, drinking within the legal driving limit, the number of units in a given drink, and the amount of calories in a drink. If anything, the present research suggests that participants were more accurate at judging the alcohol and calorie content of drinks labeled with verbal and numerical descriptors denoting lower strength than drinks denoting regular (average) strength. However, the diverging findings on self-reported understanding of strength and actual consumption suggest that, although lower strength alcohol labeling may improve explicit understanding of the content of alcohol drinks, this improved understanding may not translate into actual reduced consumption of alcohol, most likely due to self-licensing processes as described earlier.

### Strengths and Limitations

This is the first study to examine the impact of lower strength alcohol labels on consumption. Measuring a behavioral outcome is one of the main strengths of this study. The study is further strengthened by following the principles of randomized controlled trials and sampling weekly wine and beer drinkers from a representative panel of the English population. Furthermore, the study was conducted in a bar lab setting mimicking a real bar, lending face validity to the main outcome. However, as noted above the experimental setting is also a limitation of the study, since we had to prioritize experimental control over ecological validity. Moreover, the study only measured consumption over a limited time period under the pretext of a taste test of new alcohol products coming on the market. Future studies should examine more long-term effects of lower strength alcohol labeling, employing longitudinal designs, as well as replicating the current findings in real world settings.

A further limitation is the single-item nature of some of the secondary outcomes that gauged participants’ understanding of alcohol strength and calorie content. Due to time constraints and to minimize participant burden, we were unable to use multi-item scales for these secondary outcomes. Future studies could usefully extend the present findings with more extensive measurement of the constructs of interest. Furthermore, even though our sample was sampled from a nationally representative English panel, there was a sizable number of eligible participants who did not schedule a testing appointment (1,722 out of 2,118) highlighting the potential for a selection bias in the final sample.

### Conclusions

These results suggest that labeling drinks as lower in strength increases the amount consumed. Further studies are warranted to test for replication in non-laboratory settings and to estimate the potential for any effects to be at a level with the potential to harm health.

## Supplementary Material

10.1037/hea0000622.supp

## Figures and Tables

**Table 1 tbl1:** Participant Demographic and Drinking Characteristics

	Super low	Low	Regular
	*n* (%)	*n* (%)	*n* (%)
Sample size	88 (33.3)	88 (33.3)	88 (33.3)
Gender			
Male	44 (50)	44 (50)	44 (50)
Female	44 (50)	44 (50)	44 (50)
Age group			
18–44	44 (50)	44 (50)	44 (50)
45–70	44 (50)	44 (50)	44 (50)
Social grade			
Low	30 (34)	29 (33)	29 (32)
Medium	28 (32)	29 (33)	31 (35)
High	30 (34)	30 (34)	28 (33)
Ethnicity			
White	64 (72.7)	60 (68.2)	58 (65.9)
Other	23 (26.1)	28 (31.8)	30 (34.1)
NA	1 (1.1)	0	0
Education^a^			
Up to 4 GCSEs	7 (8)	8 (9.1)	10 (11.4)
1 A Level	14 (15.9)	12 (13.6)	8 (9.1)
2+ A Levels	15 (17)	13 (14.8)	17 (19.3)
University	47 (53.4)	54 (61.4)	49 (55.7)
NA	5 (5.7)	1 (1.1)	4 (4.5)
Income^b^			
[0, 15.5 K] p.a.	5 (5.7)	9 (10.2)	6 (6.8)
[15.5 K, 25.5 K] p.a.	9 (10.2)	12 (13.6)	10 (11.4)
[25 K, 40 K] p.a.	27 (30.7)	16 (18.2)	25 (28.4)
[>40 K] p.a.	47 (53.4)	51 (58)	47 (53.4)
Index of Multiple Deprivation (IMD)^c^			
Quintile 1	16 (18.2)	16 (18.2)	18 (20.5)
Quintile 2	29 (33)	18 (20.5)	17 (19.3)
Quintile 3	22 (25)	23 (26.1)	18 (20.5)
Quintile 4	10 (11.4)	9 (10.2)	16 (18.2)
Quintile 5	8 (9.1)	7 (8)	8 (9.1)
NA	3 (3.4)	15 (17)	11 (12.5)
Risky drinking (AUDIT-C)			
Mean (*SD*)	4.99 (1.94)	4.80 (1.86)	5.18 (1.75)
Taste test duration			
Mean (*SD*)	8.07 (1.41)	7.92 (1.63)	7.75 (1.54)
^a^ GCSEs (General Certificate of Secondary Education) are usually taken at age 15–16 in the UK; A-Levels at age 17–18. ^b^ Income bands are expressed per annum. ^c^ Index of Multiple Deprivation (IMD) denotes neighbourhood-level deprivation; Quintile 1 reflects the highest level of deprivation and Quintile 5 the lowest level of deprivation.			

**Table 2 tbl2:** Linear Regression Model on Total Consumption With Linear Trend of Label Groups

Variable	B	Standard error	Sig.	95% CIs
Intercept	11.33	.31	<.001	[10.72, 11.94]
Label group (linear trend)	.71	.30	.015	[.13, 1.30]
Drink type (dummy)	3.77	.50	<.001	[2.77, 4.74]

**Table 3 tbl3:** Linear Regression Model on Total Consumption With Contrasts Between Label Groups

Variable	B	Standard error	Sig.	95% CIs
Intercept	10.66	.50	<.001	[9.68, 11.63]
Super low vs. Regular (dummy)	1.43	.61	.019	[.24, 2.61]
Low vs. Regular (dummy)	.59	.63	.340	[−.66, 1.80]
Drink type (dummy)	3.77	.51	<.001	[2.78, 4.76]
