# Supplementary material for: Impact of Lower Strength Alcohol Labeling on Consumption: A Randomized Controlled Trial
Source: Health Psychol. 2018 Apr 26;37(7):658–67. doi: 10.1037/hea0000622 (PMC6001942; doi:10.1037/hea0000622)
Supplement: Supplementary file 1 [file zg1999183532so1.pdf]

Online Supplementary Materials:

Impact of lower strength alcohol labelling on consumption: A randomised controlled trial

Milica Vasiljevic[a], Dominique-Laurent Couturier[a], Daniel Frings[b], Antony C. Moss[b], Ian P. Albery[b], & Theresa M. Marteau[a]

[a] Behaviour and Health Research Unit, Institute of Public Health, University of Cambridge, Cambridge, UK
[b] Centre for Addictive Behaviours Research, Department of Psychology, School of Applied Sciences, London South Bank University, UK

Appendix 1: Image of the bar laboratory .... p. 2
Appendix 2: Sham taste test set-up .... p. 3
Appendix 3: Taste test task instructions .... p. 4
Appendix 4: Linear regression estimates .... pp. 5-10
Appendix 5: Transformation of secondary outcomes .... pp. 11-18

Appendix 1: Image of the bar laboratory

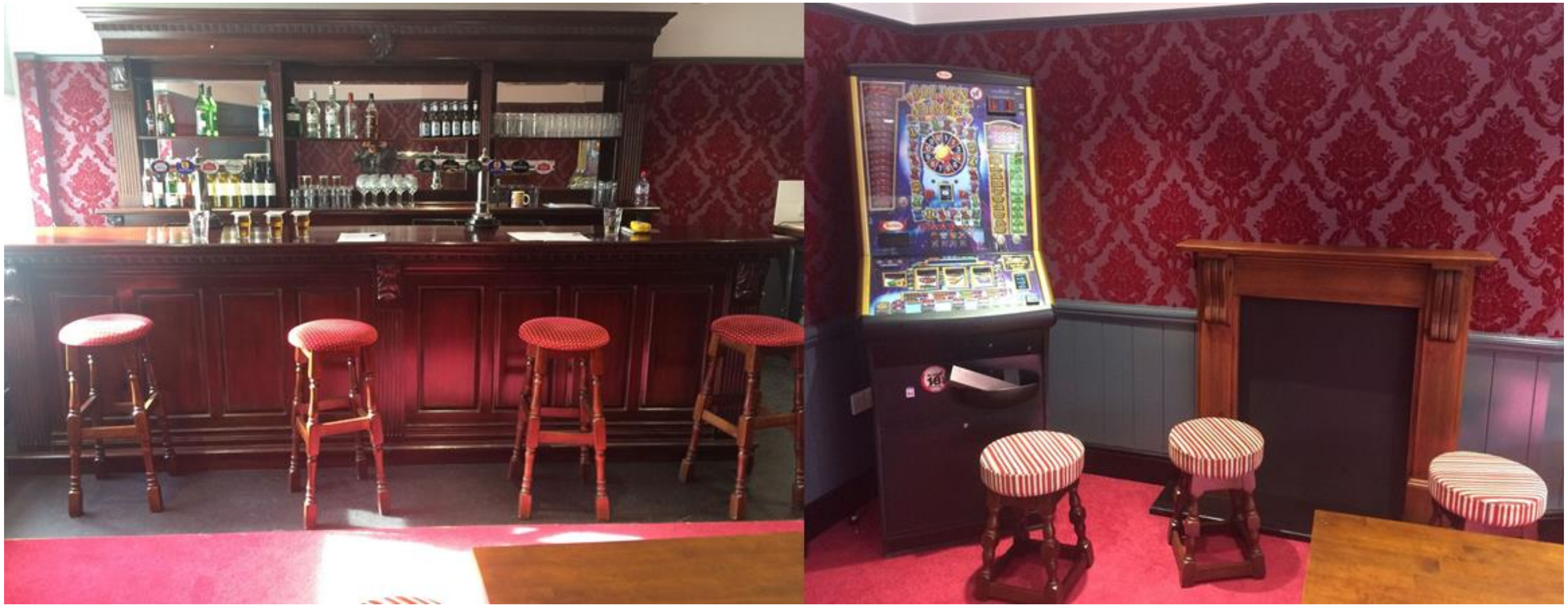

Figure S1. Image of the bar laboratory set-up.

Appendix 2: Sham taste test set-up

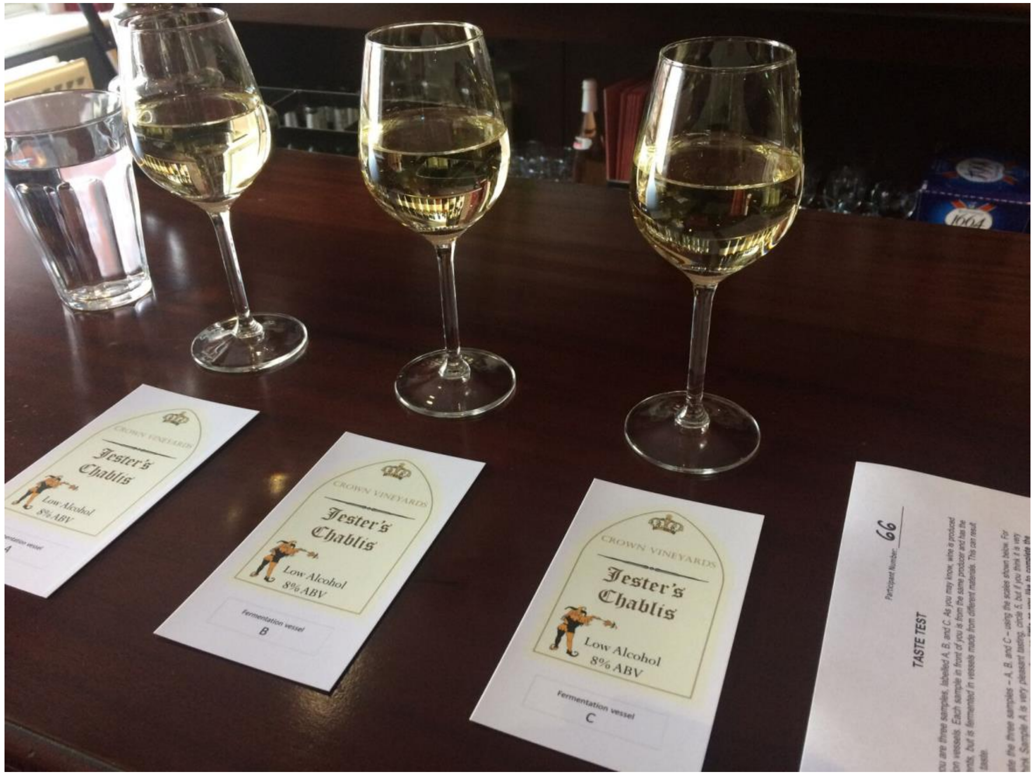

Figure S2: Sham taste test set-up in one experimental group (Group 2: *Low Alcohol 8% ABV* in wine).

Appendix 3: Taste test task instructions

TASTE TEST

In front of you are three samples, labelled A, B, and C. As you may know, wine is produced in fermentation vessels. Each sample in front of you is from the same producer and has the same ingredients, but is fermented in vessels made from different materials. This can result in variations in taste.

Your task is to rate the three samples – A, B, and C – using the scales shown below. For example, if you think Sample A is very pleasant tasting, circle 5, but if you think it is very unpleasant, circle 1. You may drink as much as you like to make your ratings.
Before you start, please have a careful look at the label to familiarise yourself with the drink.

SAMPLE A

Unpleasant	1	2	3	4	5	Pleasant
Tasteless	1	2	3	4	5	Strong tasting
Bitter	1	2	3	4	5	Sweet
Flat	1	2	3	4	5	Fizzy

SAMPLE B

Unpleasant	1	2	3	4	5	Pleasant
Tasteless	1	2	3	4	5	Strong tasting
Bitter	1	2	3	4	5	Sweet
Flat	1	2	3	4	5	Fizzy

SAMPLE C

Unpleasant	1	2	3	4	5	Pleasant
Tasteless	1	2	3	4	5	Strong tasting
Bitter	1	2	3	4	5	Sweet
Flat	1	2	3	4	5	Fizzy

Please also rank order the three samples according to your preference. In the table below, please write down your most preferred sample in the top row (i.e., "A", "B", or "C"), your least preferred sample in the bottom row, and the second most preferred sample in the middle row. Please note that you can only enter "A", "B", and "C" once, so there is clear ranking between all three samples.

	**RANK:**	**Any comments:**
Most Preferred		
Intermediate		
Least Preferred		

Appendix 4: Linear regression estimates

Table S1a.
Linear regression model on total consumption when gender, age and SES occupational status are considered in the model with linear trend of label groups.

Variable	B	Std. Error	Sig.	Sig. (global)	95% CIs
Intercept	10.14	0.46	<0.001	*	(9.25, 11.08)
Label Group (linear trend)	1.05	0.43	0.016		(0.18, 1.93)
Drink Type (dummy)	3.47	0.77	<0.001	*	(1.96, 4.98)
Gender	1.90	0.60	0.002	*	(0.74, 3.07)
Age	0.46	0.59	0.438		(-0.69, 1.62)
SES Occupational status	-0.07	0.38	0.855		(-0.82, 0.68)
Label Group (linear trend) X Gender	-0.75	0.56	0.181		(-1.85, 0.36)
Drink Type (dummy) X Gender	2.30	0.94	0.015		(0.48, 4.17)
Label Group (linear trend) X Age	0.08	0.55	0.889		(-0.99, 1.17)
Drink Type (dummy) X Age	-1.67	0.93	0.076		(-3.50, 0.15)
Label Group (linear trend) X SES Occupational status	0.28	0.35	0.411		(-0.40, 0.98)
Drink Type (dummy) X SES Occupational status	-0.02	0.57	0.976		(-1.17, 1.11)

Note. Global significance level ($p < .05$) when correcting for multiple comparisons with the Holm-Šídák correction. Only effect estimates denoted with * in the column Sig (global) are significant when correcting for multiple comparisons.

Table S1b.

Linear regression model on total consumption when gender, age and SES occupational status are considered in the model with contrasts between label groups.

Variable	B	Std. Error	Sig.	Sig. (global)	95% CIs
Intercept	9.04	0.78	<0.001	*	(7.55, 10.57)
Super Low vs. Regular (dummy)	2.11	0.87	0.016		(0.34, 3.79)
Low vs. Regular (dummy)	1.15	1.01	0.256		(-0.86, 3.10)
Drink Type (dummy)	3.47	0.79	<0.001	*	(1.95, 4.98)
Gender	2.73	0.94	0.004	*	(0.86, 4.58)
Age	0.54	0.94	0.573		(-1.23, 2.39)
SES Occupational status	0.22	0.58	0.710		(-0.90, 1.38)
Super Low vs. Regular (dummy) X Gender	-1.51	1.09	0.176		(-3.58, 0.69)
Low vs. Regular (dummy) X Gender	-0.80	1.14	0.486		(-3.01, 1.44)
Drink Type (dummy) X Gender	2.25	0.92	0.017		(0.41, 4.06)
Super Low vs. Regular (dummy) X Age	0.14	1.09	0.896		(-2.06, 2.25)
Low vs. Regular (dummy) X Age	-0.30	1.14	0.801		(-2.54, 1.86)
Drink Type (dummy) X Age	-1.68	0.95	0.080		(-3.53, 0.18)
Super Low vs. Regular (dummy) X SES Occupational status	0.53	0.68	0.432		(-0.82, 1.86)
Low vs. Regular (dummy) X SES Occupational status	-1.39	0.68	0.041		(-2.75, -0.07)
Drink Type (dummy) X SES Occupational status	-0.02	0.56	0.974		(-1.12, 1.08)

Note. Global significance level ($p < .05$) when correcting for multiple comparisons with the Holm-Šídák correction. Only effect estimates denoted with * in the column Sig (global) are significant when correcting for multiple comparisons.

Table S2a.

Linear regression model on total consumption when gender, age, SES occupational status, SES education, SES income, SES Index of Multiple Deprivation, risky drinking, motivation to reduce consumption, and self-licensing are considered in the model with linear trend of label groups.

Variable	B	Std. Error	Sig. (2-tailed)	Sig. (global)	95% CIs
Intercept	8.40	0.87	<0.001	*	(6.58, 10.01)
Label Group (linear trend)	2.23	0.80	0.004		(0.72, 3.80)
Drink Type (dummy)	4.03	1.40	0.004		(1.48, 6.93)
Gender	1.47	0.66	0.027		(0.25, 2.80)
Age	1.08	0.67	0.107		(-0.21, 2.40)
SES Occupational status	-0.50	0.41	0.225		(-1.35, 0.26)
Education	-0.02	0.33	0.958		(-0.62, 0.67)
Income	0.25	0.38	0.502		(-0.48, 1.02)
IMD Rank (area deprivation)	-0.16	0.30	0.579		(-0.76, 0.40)
AUDIT-C (binary)	2.46	0.72	0.001	*	(1.00, 3.83)
Self-Licensing	-0.04	0.25	0.884		(-0.52, 0.45)
Motivation to Reduce Consumption	0.17	0.20	0.385		(-0.21, 0.58)
Label Group (linear trend) X Gender	-1.06	0.62	0.084		(-2.34, 0.14)
Drink Type (dummy) X Gender	2.77	1.01	0.006		(0.73, 4.67)
Label Group (linear trend) X Age	-0.50	0.59	0.389		(-1.60, 0.73)
Drink Type (dummy) X Age	-1.59	1.07	0.138		(-3.69, 0.51)
Label Group (linear trend) X SES Occupational status	0.69	0.38	0.069		(-0.07, 1.45)
Drink Type (dummy) X SES Occupational status	-0.24	0.66	0.708		(-1.48, 1.07)
Label Group (linear trend) X Education	-0.20	0.32	0.521		(-0.85, 0.41)

Drink Type (dummy) X Education	-0.12	0.54	0.818		(-1.23, 0.87)
Label Group (linear trend) X Income	-0.44	0.39	0.234		(-1.21, 0.32)
Drink Type (dummy) X Income	0.08	0.63	0.899		(-1.21, 1.29)
Label Group (linear trend) X IMD Rank (area deprivation)	-0.51	0.28	0.061		(-1.06, 0.03)
Drink Type (dummy) X IMD Rank (area deprivation)	0.03	0.44	0.941		(-0.79, 0.93)
Label Group (linear trend) X AUDIT-C (binary)	-0.61	0.68	0.366		(-2.02, 0.67)
Drink Type (dummy) X AUDIT-C (binary)	-1.18	1.14	0.299		(-3.36, 1.12)
Label Group (linear trend) X Self-Licensing	0.39	0.23	0.084		(-0.06, 0.83)
Drink Type (dummy) X Self-Licensing	-0.31	0.39	0.431		(-1.03, 0.52)
Label Group (linear trend) X Motivation to Reduce Consumption	0.07	0.20	0.711		(-0.31, 0.48)
Drink Type (dummy) X Motivation to Reduce Consumption	-0.74	0.33	0.030		(-1.46, -0.14)

Note. Global significance level ($p < .05$) when correcting for multiple comparisons with the Holm-Šídák correction. Only effect estimates denoted with * in the column Sig (global) are significant when correcting for multiple comparisons.

Table S2b.

Linear regression model on total consumption when gender, age, SES occupational status, SES education, SES income, SES Index of Multiple Deprivation, risky drinking, motivation to reduce consumption, and self-licensing are considered in the model with contrasts between label groups.

Variable	B	Std. Error	Sig.	Sig. (global)	95% CIs
Intercept	6.07	1.40	<0.001	*	(3.07, 8.64)
Super Low vs. Regular (dummy)	4.67	1.66	0.004		(1.62, 8.10)
Low vs. Regular (dummy)	2.07	1.92	0.271		(-1.68, 5.99)
Drink Type (dummy)	4.04	1.53	0.010		(1.32, 7.29)
Gender	2.54	1.16	0.028		(0.31, 4.90)
Age	1.60	1.06	0.130		(-0.60, 3.57)
SES Occupational status	-0.86	0.70	0.216		(-2.21, 0.52)
Education	0.09	0.61	0.880		(-1.05, 1.32)
Income	0.58	0.63	0.340		(-0.61, 1.92)
IMD Rank (area deprivation)	0.29	0.51	0.558		(-0.79, 1.22)
AUDIT-C (binary)	3.40	1.20	0.006		(1.10, 5.76)
Self-Licensing	-0.61	0.41	0.140		(-1.43, 0.19)
Motivation to Reduce Consumption	0.11	0.28	0.676		(-0.42, 0.71)
Super Low vs. Regular (dummy) X Gender	-2.14	1.28	0.096		(-4.68, 0.39)
Low vs. Regular (dummy) X Gender	-0.57	1.51	0.709		(-3.45, 2.41)
Drink Type (dummy) X Gender	2.53	1.08	0.019		(0.41, 4.66)
Super Low vs. Regular (dummy) X Age	-1.07	1.18	0.365		(-3.30, 1.37)
Low vs. Regular (dummy) X Age	-0.61	1.43	0.667		(-3.40, 2.21)
Drink Type (dummy) X Age	-1.39	1.13	0.218		(-3.63, 0.84)
Super Low vs. Regular (dummy) X SES Occupational status	1.37	0.77	0.077		(-0.17, 2.90)
Low vs. Regular (dummy) X SES Occupational	-0.62	0.86	0.458		(-2.37, 1.01)

status				
Drink Type (dummy) X SES Occupational status	-0.20	0.67	0.756	(-1.46, 1.15)
Super Low vs. Regular (dummy) X Education	-0.41	0.67	0.529	(-1.78, 0.83)
Low vs. Regular (dummy) X Education	0.28	0.81	0.728	(-1.36, 1.84)
Drink Type (dummy) X Education	-0.06	0.61	0.920	(-1.36, 1.06)
Super Low vs. Regular (dummy) X Income	-0.87	0.81	0.272	(-2.46, 0.76)
Low vs. Regular (dummy) X Income	-0.12	0.76	0.865	(-1.64, 1.36)
Drink Type (dummy) X Income	0.01	0.68	0.982	(-1.37, 1.29)
Super Low vs. Regular (dummy) X IMD Rank (area deprivation)	-0.92	0.57	0.103	(-2.02, 0.19)
Low vs. Regular (dummy) X IMD Rank (area deprivation)	-0.37	0.58	0.516	(-1.48, 0.81)
Drink Type (dummy) X IMD Rank (area deprivation)	-0.01	0.50	0.977	(-0.90, 1.08)
Super Low vs. Regular (dummy) X AUDIT-C (binary)	-1.36	1.40	0.334	(-4.27, 1.22)
Low vs. Regular (dummy) X AUDIT-C (binary)	-1.59	1.60	0.318	(-4.95, 1.43)
Drink Type (dummy) X AUDIT-C (binary)	-1.19	1.20	0.315	(-3.58, 1.09)
Super Low vs. Regular (dummy) X Self-Licensing	0.80	0.47	0.089	(-0.10, 1.76)
Low vs. Regular (dummy) X Self-Licensing	0.93	0.51	0.066	(-0.06, 1.94)
Drink Type (dummy) X Self-Licensing	-0.23	0.41	0.570	(-1.03, 0.58)
Super Low vs. Regular (dummy) X Motivation to Reduce Consumption	0.12	0.40	0.759	(-0.65, 0.96)
Low vs. Regular (dummy) X Motivation to Reduce Consumption	0.24	0.40	0.540	(-0.57, 1.01)
Drink Type (dummy) X Motivation to Reduce Consumption	-0.78	0.34	0.022	(-1.48, -0.15)

Note. Global significance level ($p < .05$) when correcting for multiple comparisons with the Holm-Šídák correction. Only effect estimates denoted with * in the column Sig (global) are significant when correcting for multiple comparisons.

Appendix 5: Transformations/calculations of variables

Item 1- level of understanding as assessed for level of perceived appropriateness for children to consume given drink:

The question was worded as: "**This wine/beer can be safely drunk by children aged over 12. Do you agree with this statement?". Responses were recorded on a scale from 1 = strongly disagree to 7 = strongly agree.**

Participants' responses were dichotomised whereby any level of disagreement with the statement was considered correct, and any level of agreement as incorrect. This dichotomised variable was then used in logistic regression analysis. A graphical presentation of the results is given below.

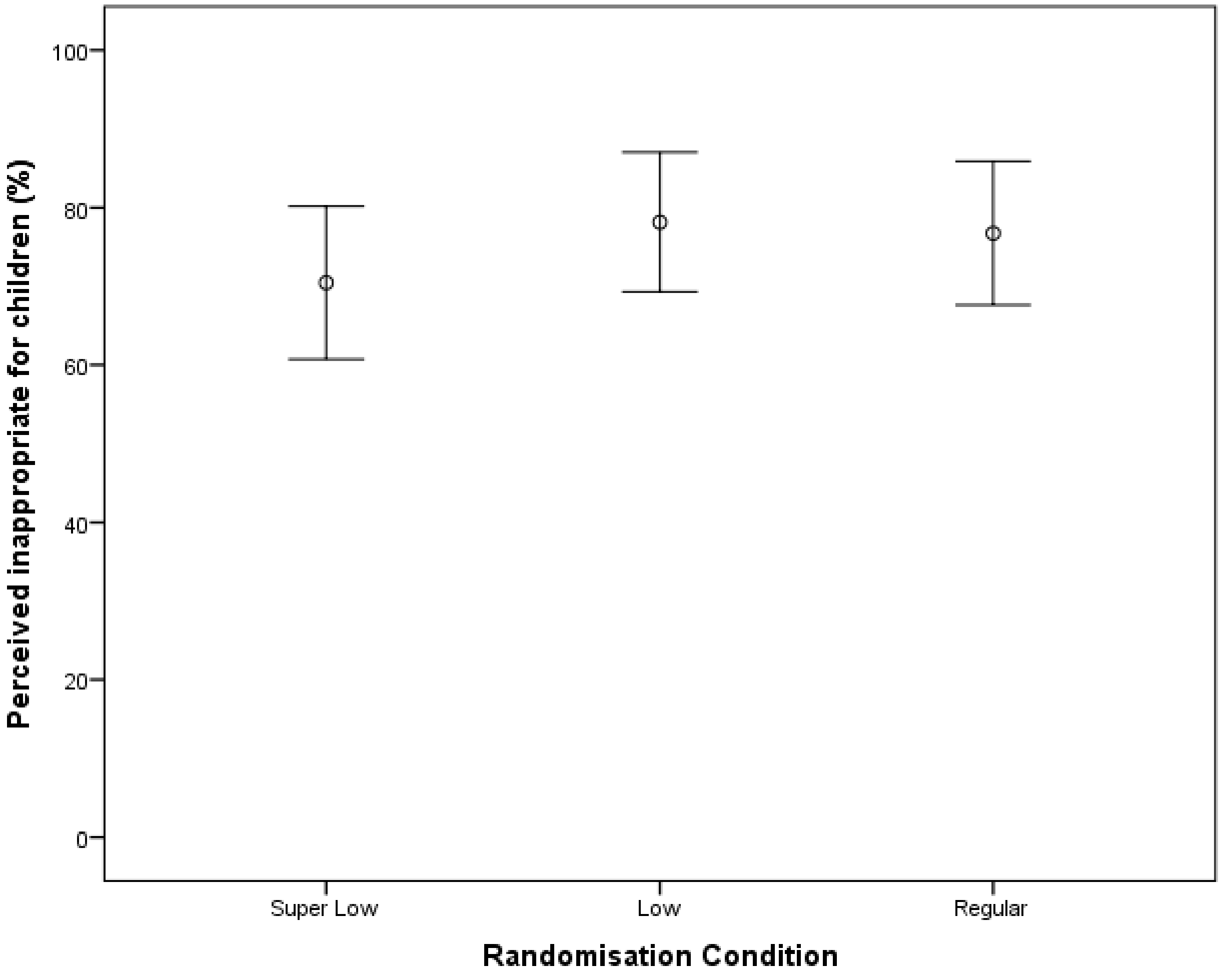

Figure S3. Proportion of participants perceiving a drink with a given label as inappropriate for consumption by children.

Item 2 – level of understanding as assessed for knowledge of drinks suitable for driving within the legal limit:

The question was worded as: **How many <u>small glasses (125 ml)</u> of this wine/<u>half-pints</u> of this beer do you think you could have and still drive within the legal limit? [0-20 scale on a slider]**

We first ascertained the current drink-driving limit in the UK excluding Scotland (https://www.gov.uk/drink-drive-limit):

**Level of alcohol**	England, Wales and Northern Ireland
Micrograms per 100 millilitres of breath	35
Milligrammes per 100 millilitres of blood	80
Milligrammes per 100 millilitres of urine	107

We then calculated the blood alcohol content for an average male and female if they consumed a small glass (125ml)/half-pint of a wine/beer with a given %ABV. For the different BAC calculations in wine and beer see Table S3a and S3b below.

Table S3a & b: BAC for an average male and female after consuming a small glass of wine/half-pint of beer.

**Wine (% ABV)**	**Male (125ml BAC)**	**Female (125ml BAC)**
Super Low [4%]	0.007	0.009
Low [8%]	0.013	0.019
No% No Verbal Label [12.9%]	0.021	0.03

**Beer (% ABV)**	**Male (1/2 pint BAC)**	**Female (1/2 pint BAC)**
Super Low [1%]	0.004	0.005
Low [3%]	0.011	0.016
No% No Verbal Label [4.2%]	0.016	0.022

Taking into account the legal drink-driving limit in the UK (excl. Scotland) we then worked out the correct answer of how many small glasses (125ml)/half-pints of wine/beer with a given %ABV one could consume and still drive with the legal limit. These figures are shown in the Table S4a & S4b below.

Table S4a & b: Limit of small glasses of wine/half-pints of beer one can drink and still drive within the legal limit.

**Wine (% ABV)**	**Male (No. 125ml)**	**Female (No. 125ml)**
Super Low [4%]	11	8
Low [8%]	6	4
No% No Verbal Label [12.9%]	3	2

Beer (% ABV)	Male (No. 1/2 pints)	Female (No. 1/2 pints)
Super Low [1%]	20	16
Low [3%]	7	5
No% No Verbal Label [4.2%]	5	3

We then dichotomised the variable so that we categorised together the proportion of people answering correctly or under-estimating the number of drinks versus those over-estimating the number of drinks one could have and still drive within the limit. This dichotomized variable was then used for logistic regression analyses, which are graphically shown below.

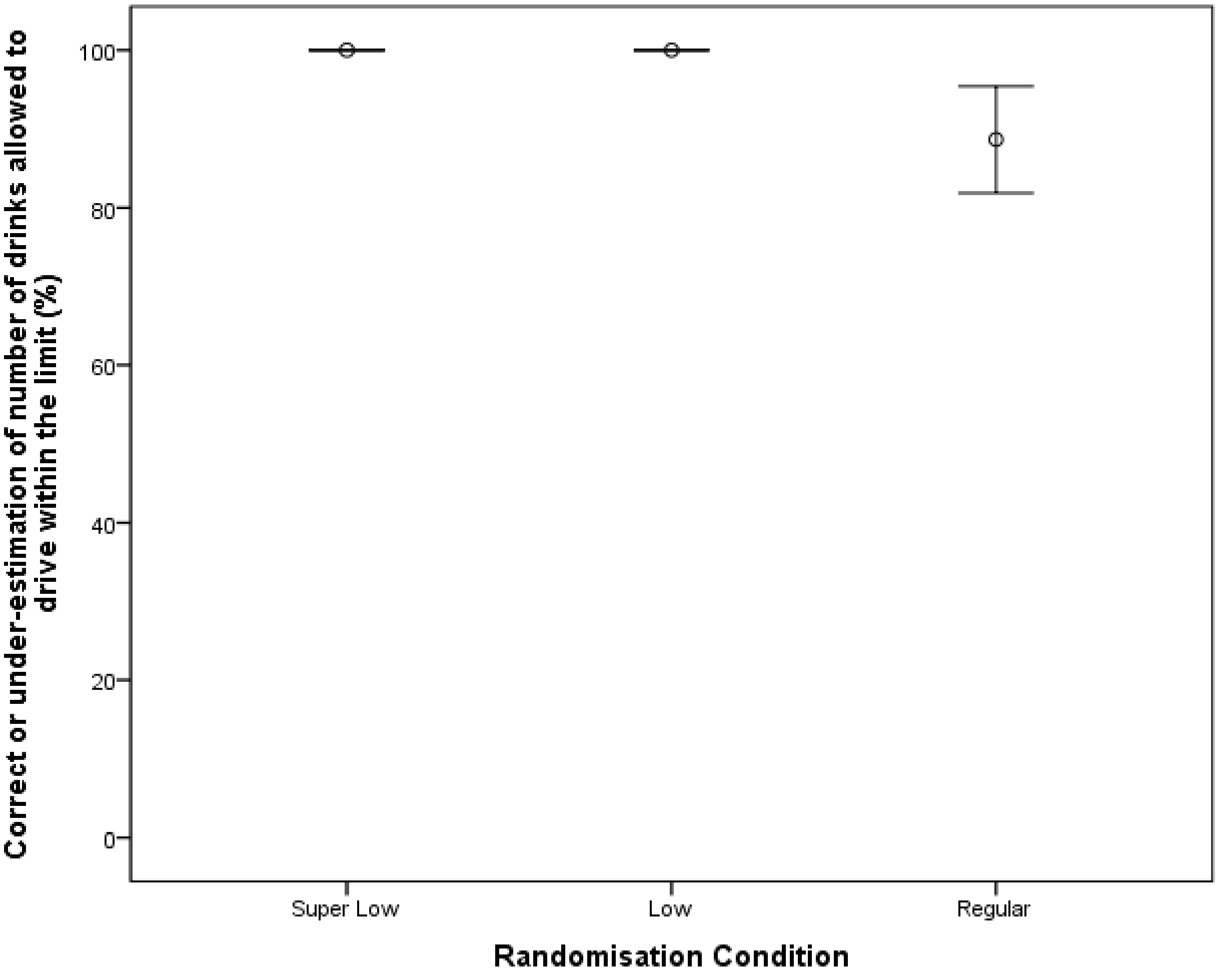

Figure S4. Proportion of participants correctly identifying or under-estimating the number of drinks they could have with a given label and still drive within the legal limit.

Item 3 – understanding of units contained in a small glass (125ml)/half pint of a given drink:

The question was worded as: **“How many units of alcohol do you think a small glass (125ml)/half-pint of this wine/beer would have?” [0-20 slider scale]**

We first calculated the actual units contained in each of the drinks according to its %ABV. The formula used for these calculations was strength (ABV) x volume (ml) ÷ 1,000 = units. The below tables show how many units are contained in a small glass (125ml) of wine or half-pint of beer with the different %ABV used in the study design. We then used these figures to calculate whether participant’s responses were correct, underestimation or overestimation of the correct figure (see Table 5a & b below).

Table S5a & b: Units of alcohol contained in a small glass of wine/half-pint of beer.

**Wine (% ABV)**	**Units in 125ml**
Super Low [4%]	0.5
Low [8%]	1
No% No Verbal Label [12.9%]	1.6

**Beer (% ABV)**	**Units in 1/2 pint**
Super Low [1%]	0.3
Low [3%]	0.9
No% No Verbal Label [4.2%]	1.2

For each experimental condition we then determined the proportion of people who answered correctly, proportion who under-estimated, and proportion who over-estimated. The under-estimators would be the ones we would be concerned about, hence we categorised the correct answers and over-estimations versus the under-estimations. We then performed logistic regression, which are graphically shown below.

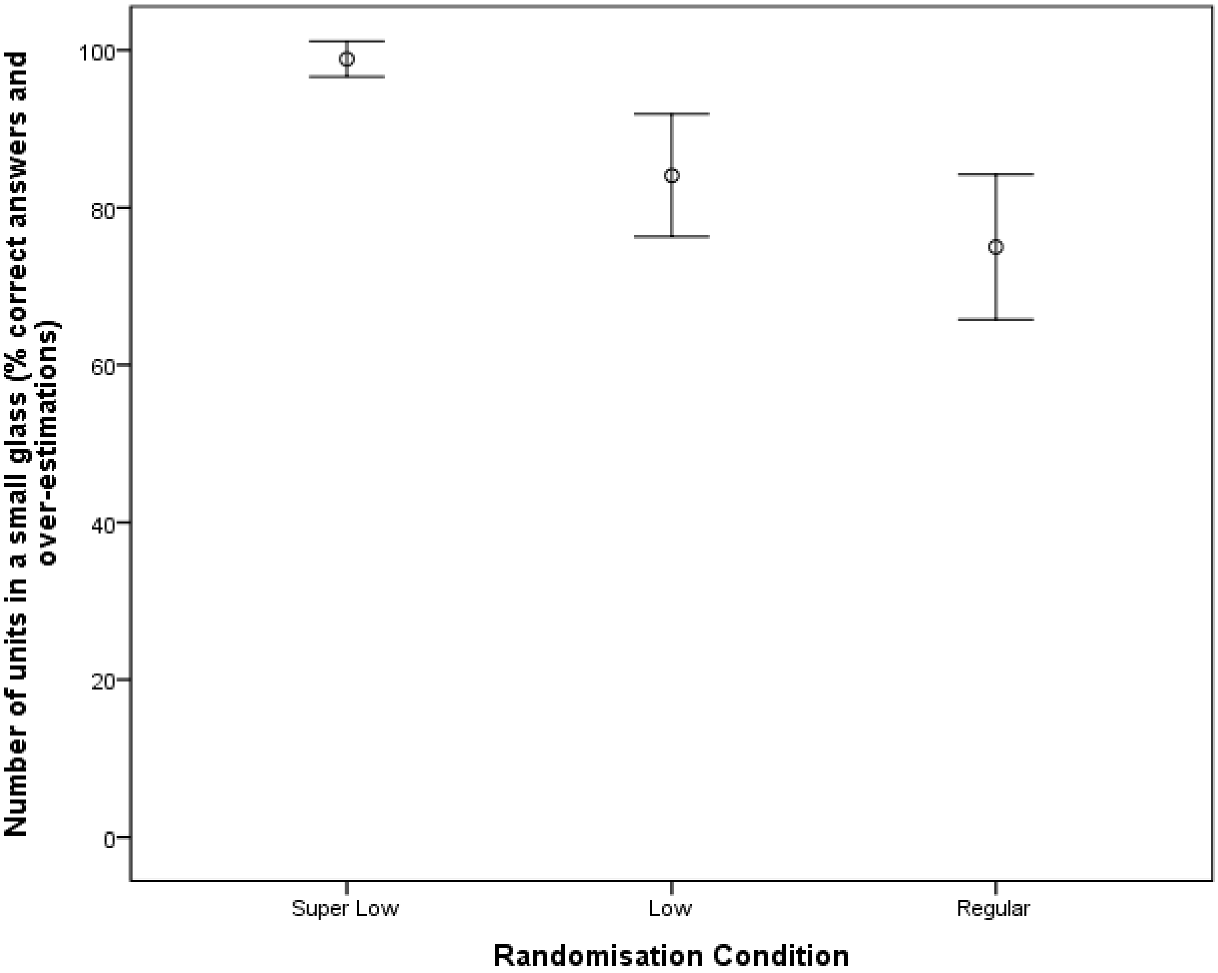

Figure S5. Proportion of participants correctly identifying or over-estimating the number of units in a drink with a given label.

Calorie content estimation:

Assessed by one item: **"The recommended daily calorie intake from food and drinks for men is 2500 Calories (kcal), and for women 2000 Calories (kcal). How many Calories (kcal) do you think a half-pint of this beer has?" [Responses were open-ended, but constrained to responses ranging from 0-2500]**

We worked out the correct answer presented in the table below for each of the %ABV conditions. We used the following formula for these calculations:

$$\frac{\text{volume (ml) x alcohol (ABV \%) x 8}}{1000}$$

Multiplying this answer by seven gives the approximate calorie content.
Calories from Carbohydrates (sugar): 4 calories per gram.

Table S6a & b: Calories in a small glass of wine/half-pint of beer.

	Regular		
Volume (ml)	125	125	125
%ABV	12.9	8	4
Calories from %ABV	90.3	56	28
Calories from Sugar	15	15	15
**Calories Total**	**105.3**	**71**	**43**

	Regular	Low	
Volume (ml)	284.131	284.131	284.131
%ABV	4.2	3	1
Calories from %ABV	66.82761	47.73401	15.91134
Calories from Sugar	40.22158	40.22158	40.22158
**Calories Total**	**107.0492**	**87.95559**	**56.13292**

For each experimental condition we determined the proportion of people who answered correctly, proportion who under-estimated, and proportion who over-estimated the calorie content. The under-estimators would be the ones we would be concerned about. Hence, we transformed the variable by categorising the correct answers with the over-estimations versus the under-estimations. This transformed dichotomous variable was then subjected to logistic regression analysis (see graph below).

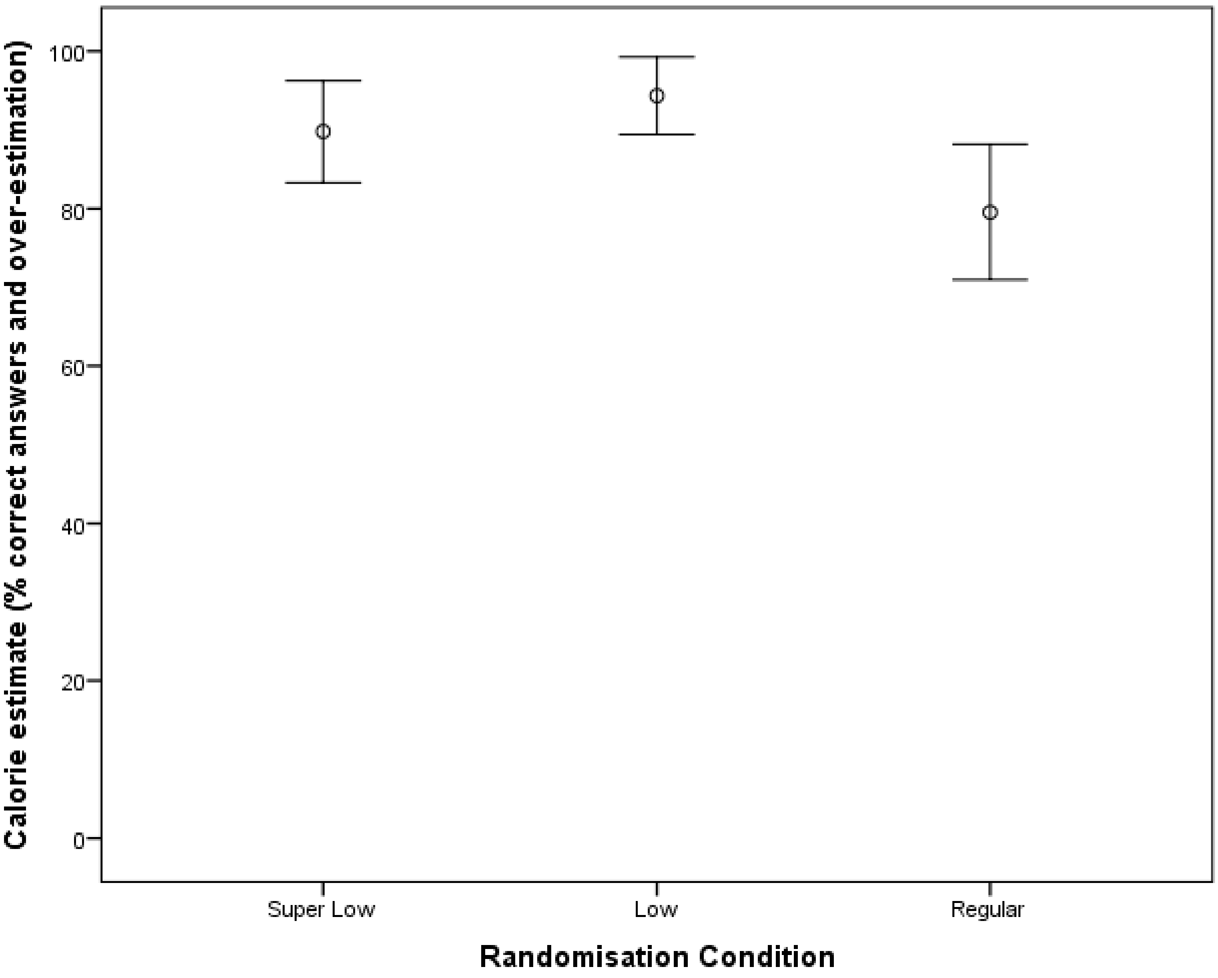

Figure S6. Proportion of participants correctly identifying or over-estimating the number of calories in a drink with a given label.

Guilt associated with consumption:

One item based on Wansink and Chandon (2006): **"How guilty would you feel after consuming a small glass (125ml)/half-pint of this wine/beer?". Answers were recorded on scales from 1 = Not Guilty to 9 = Guilty.**

We first dichotomised the variable into those not guilty versus those who reported they would feel guilty after consuming one small glass of the drink in front of them. We then carried out logistic regressions on this variable (see graph).

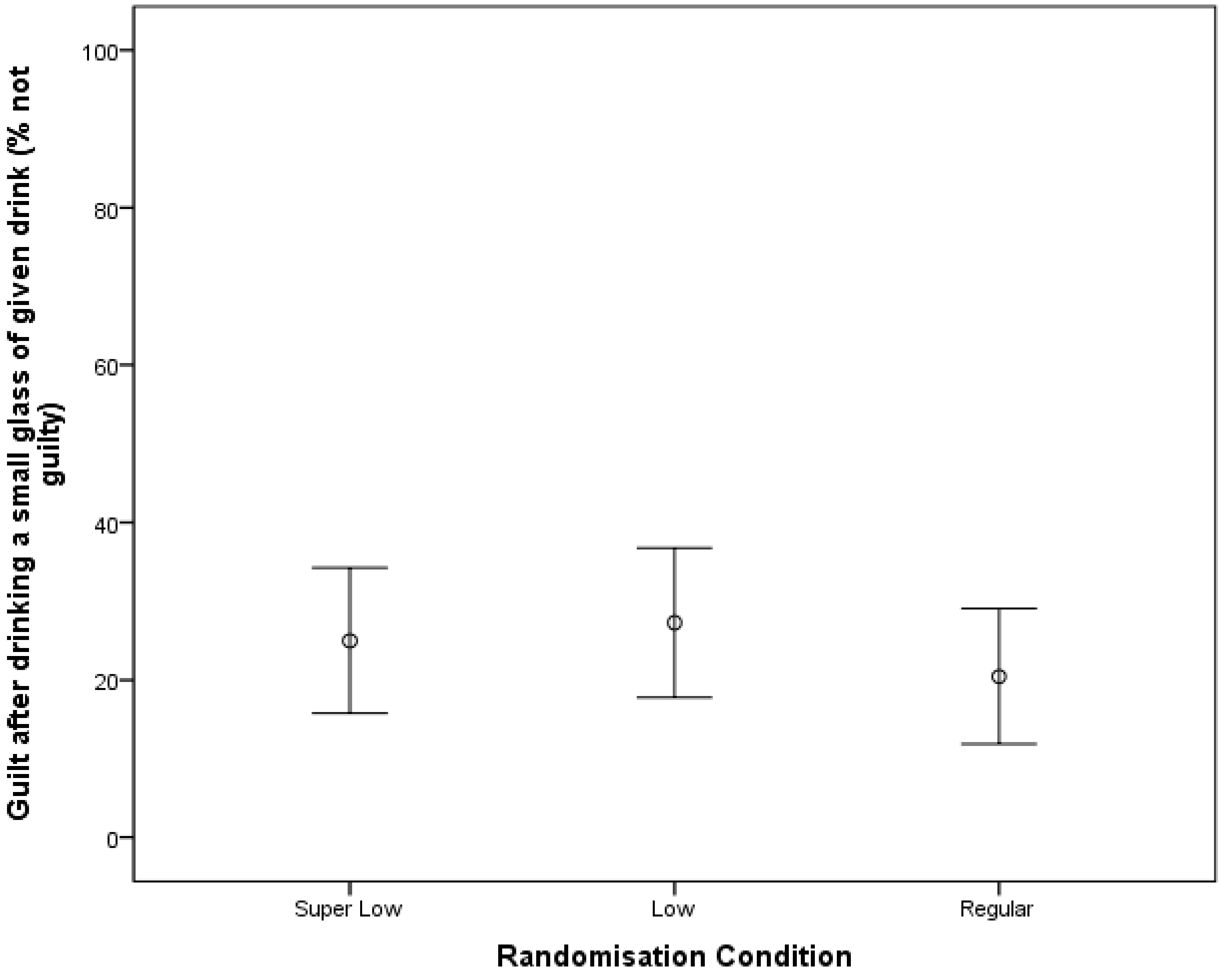

Figure S7. Proportion of participants feeling no guilt after consuming a drink with a given label.

In order to follow analyses reported by Wansink and Chandon (2006) we also explored interactions between risky drinking and low/er vs. regular labels on guilt. This yielded no significant effects. Furthermore, we also examined the three-way interaction between risky drinking, low/er *vs.* regular labels, and guilt as predictors of the primary outcome - consumption. There were no significant effects.
